# Morphological and molecular characterization of *Labronema montanum* sp. n. (Dorylaimida, Dorylaimidae) from Spain

**DOI:** 10.21307/jofnem-2019-029

**Published:** 2019-06-03

**Authors:** R. Peña-Santiago, J. Abolafia

**Affiliations:** 1 Departamento de Biología Animal, Biología Vegetal y Ecología, Universidad de Jaén, Campus “Las Lagunillas” s/n, 23071-Jaén, Spain

**Keywords:** Description, Dorylaims, *Labronema*, LSU, Morphology, New species, SEM, Taxonomy

## Abstract

A new species of the genus *Labronema*, collected in a natural mountain habitat of the southern Iberian Peninsula, in Spain, is studied, including its morphological and morphometric characterization, SEM observations, and D2-D3 28S-rRNA sequences. *Labronema montanum* sp. n. is distinguishable by its 1.56 to 2.08 mm long body, with lip region being offset by constriction and 19 to 24 µm broad, odontostyle 21 to 29 µm long, neck 417 to 551 µm long, pharyngeal expansion 205 to 272 µm long, the presence of three cardiac lobes at the pharyngo-intestinal junction, a long and tripartite uterus, longitudinal vulva (*V* = 57-60), a short and rounded caudal region (19–35 µm, *c* = 56-86, *c* = 0.5-0.8), spicules 65 to 76 µm long, and 20 to 25 nearly contiguous ventromedian supplements with hiatus.

The genus *Labronema* Thorne, 1939 is a worldwide dorylaimid (order Dorylaimida) group with about 41 valid species of intricate taxonomy (cf. Andrássy, 2009) and predator feeding habits (Thorne, 1939, 1974). The study of its representatives in the Iberian fauna has not received too much attention, except for the detailed description of *L. vulvapapillatum* (Meyl, 1954) Loof & Grootaert, 1981 by Murillo-Navarro and Jiménez-Guirado (2006; see also Jiménez-Guirado, 1989); the available information is nearly restricted to reporting the presence of other seven species (Peña-Santiago et al., 2003).

A nematological survey conducted in November 2017 to explore the dorylaimid diversity in a mountain area of the southern Iberian Peninsula yielded a *Labronema* population consisting of numerous females and males. An additional study revealed that it was an unknown species of the genus that is described herein.

## Material and methods

### Nematode extraction and processing

Nematodes were collected from a grassy area with stony soil at 1,800 elevation in Pandera mountain, Jaén province, southern Iberian Peninsula, Spain. They were extracted using Flegg’s (1967) sieving method and a modified Baermann’s (1917) funnel technique; they were killed with heat, fixed into 4% formalin, transferred to pure glycerine following Siddiqi’s (1964) method, and mounted on permanent glass slides.

### Light microscopy (LM)

Observations, measurements, line illustrations, and LM photomicrographs were made using a Nikon Eclipse 80i (Nikon, Tokio, Japan) microscope with differential interference contrast (DIC) optics, a drawing tube (*camera lucida*) and a Nikon Digital Sight DS-U1 camera. Photographs were edited using Adobe® Photoshop® CS software.

### Scanning electron microscopy (SEM)

Specimens preserved in glycerine were selected for observation under SEM according to Abolafia (2015). They were hydrated in distilled water, dehydrated in a graded ethanol–acetone series, critical point dried, coated with gold, and observed with a Zeiss Merlin microscope (5 kV) (Zeiss®, Oberkochen, Germany).

### DNA extraction, PCR, and sequencing

Nematode DNA was extracted from single fresh individuals using the proteinase K protocol and PCR assays, as described in the study of Castillo et al. (2003). The specimen was cut in small pieces using a sterilized needle on a clean slide, with 18 ml of AE buffer (10 mM Tris-Cl + 0.5 mM EDTA; pH 9.0), it was transferred to a microtube and 2 μl proteinase K (700 μg/ml−1) (Roche®, Basel, Switzerland) was added, and it was stored at −80°C for 15 min (for several days). The microtubes were incubated at 65°C for 1 hr, followed by 95°C for 15 min. The microtube was centrifuged at 13,000 r.p.m. or 15,900× g for 3 min, and 2 μl of the supernatant extracted DNA was transferred to a microtube containing 2.5 μl 10× PCR reaction buffer 5 μl Q-solution 5×, 0.5 μl dNTPs mixture (10 mM each), 1 μl of each primer (10 mM), 0.2 μl Taq DNA Polymerase (Qiagen®, Venlo, the Netherlands), and ddH2O with a final volume of 25 μl. The primers used for amplification of the D2-D3 region of 28S rRNA gene were the D2A (5′-ACAAGTACCGTGAGGGAAAGTTG-3′) and the D3B (5′-TCGGAAGGAACCAGCTACTA-3′) primers (De Ley et al., 1999). PCR cycle conditions were as follows: one cycle of 94°C for 3 min, followed by 35 cycles of 94°C for 1 min + annealing temperature of 55°C for 45 s + 72°C for 2 min, and finally one cycle of 72°C for 10 min. After DNA amplification, 5 μl of product was loaded on a 1% agarose gel in 0.5% Tris-acetate-EDTA (40 mM Tris, 20 mM glacial acetic acid and 2 mM EDTA; pH = 8) to verify the amplification using an electrophoresis system (Labnet Gel XL Ultra V-2, Progen Scientific®, London, UK). The bands were stained with RedSafe (20,000×), which was previously added to the agarose gel solution. The sequencing reactions were performed at Sistemas Genómicos (Valencia, Spain). The sequences obtained (with 769, 772 and 783 pb) were submitted to the GenBank database under accession numbers MK894244, MK894245 and MK894246.

### Phylogenetic analyses

For phylogenetic relationships, analyses were based on 28S rDNA. The newly obtained sequences were manually edited using BioEdit 7.2.6 (Hall, 1999) and aligned with another D2–D3 expansion segments of 28S rRNA gene sequences available in GenBank, using MUSCLE alignment tool implemented in the MEGA7 (Kumar et al., 2016). The ambiguously aligned parts and divergent regions were known using the online version of Gblocks 0.91b (Castresana, 2000) (http://molevol.cmima.csic.es/castresana/Gblocks_server.html) and were removed from the alignments using MEGA7. The best-fit model of nucleotide substitution used for the phylogenetic analysis was statistically selected using jModelTest 2.1.10 (Darriba et al., 2012). Phylogenetic tree was generated with Bayesian inference method using MrBayes 3.2.6 (Huelsenbeck and Ronquist, 2001; Ronquist and Huelsenbeck, 2003). *Mononchus truncatus* Bastian, 1865 (AY593064) was chosen as an outgroup. The analysis under GTR + I + G model was initiated with a random starting tree and run with the Markov Chain Monte Carlo (MCMC) for 1 × 106 generations. The tree was visualized and saved with FigTree 1.4.3 (Rambaut, 2014).

## Results

*Labronema montanum* sp. n.

(Figs. 1–4)

**Figure 1:**
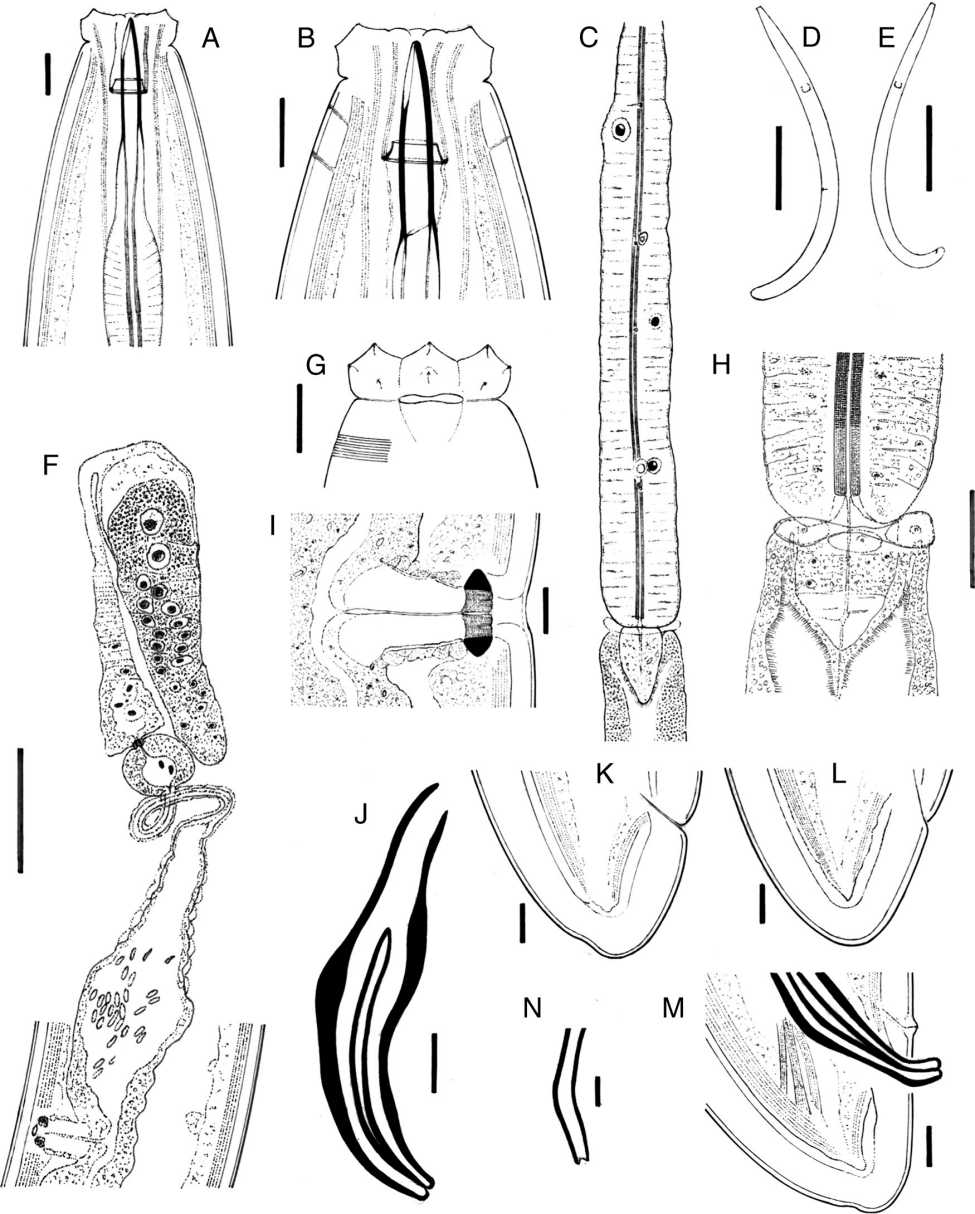
*Labronema montanum* sp. n. from Spain. (A, B): Anterior region in lateral, median view. (C): Pharyngeal expansion. (D): Female, entire. (E): Male, entire. (F): Female, anterior genital branch. (G): Lip region in lateral, surface view. (H): Pharyngo-intestinal junction. (I): Vagina. (J): Spicule. (K, L): Female tail. (M): Male tail. (N): Lateral guiding piece. (Scale bars: A, B, G, I–M = 10 µm; C, F = 50 µm; D, E = 500 µm; H = 20 µm; N = 5 µm).

**Figure 2:**
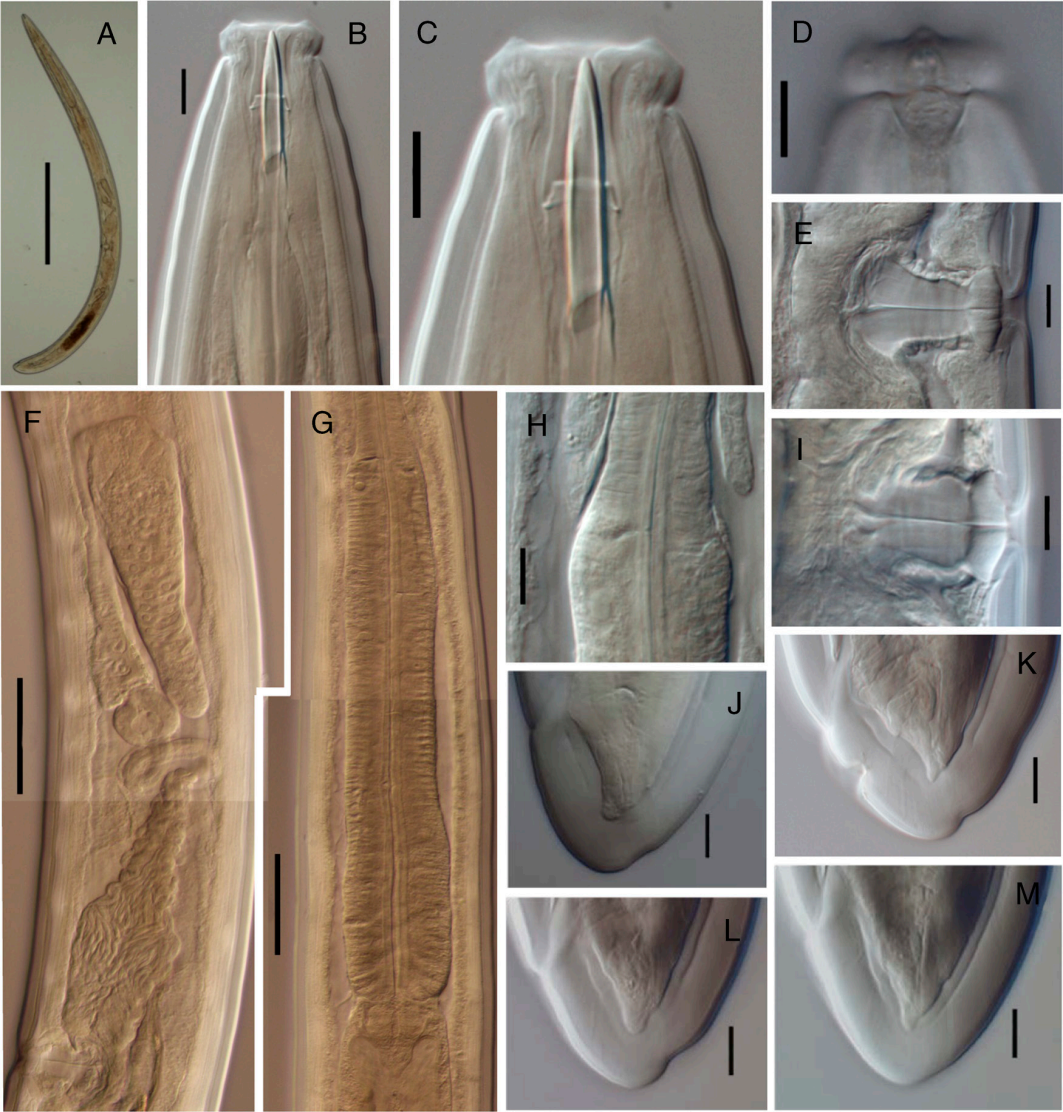
*Labronema montanum* sp. n. from Spain (female, LM). (A): Entire. (B, C): Anterior region in lateral median view. (D): Lip region in lateral, surface view. (E, I): Vagina. (F): Anterior genital branch. (G): Pharyngeal expansion. (H): Pharyngeal enlargement. (J–M): Tail. (Scale bars: A = 500 µm; B–D, H–M = 10 µm; F, G = 50 µm.)

**Figure 3:**
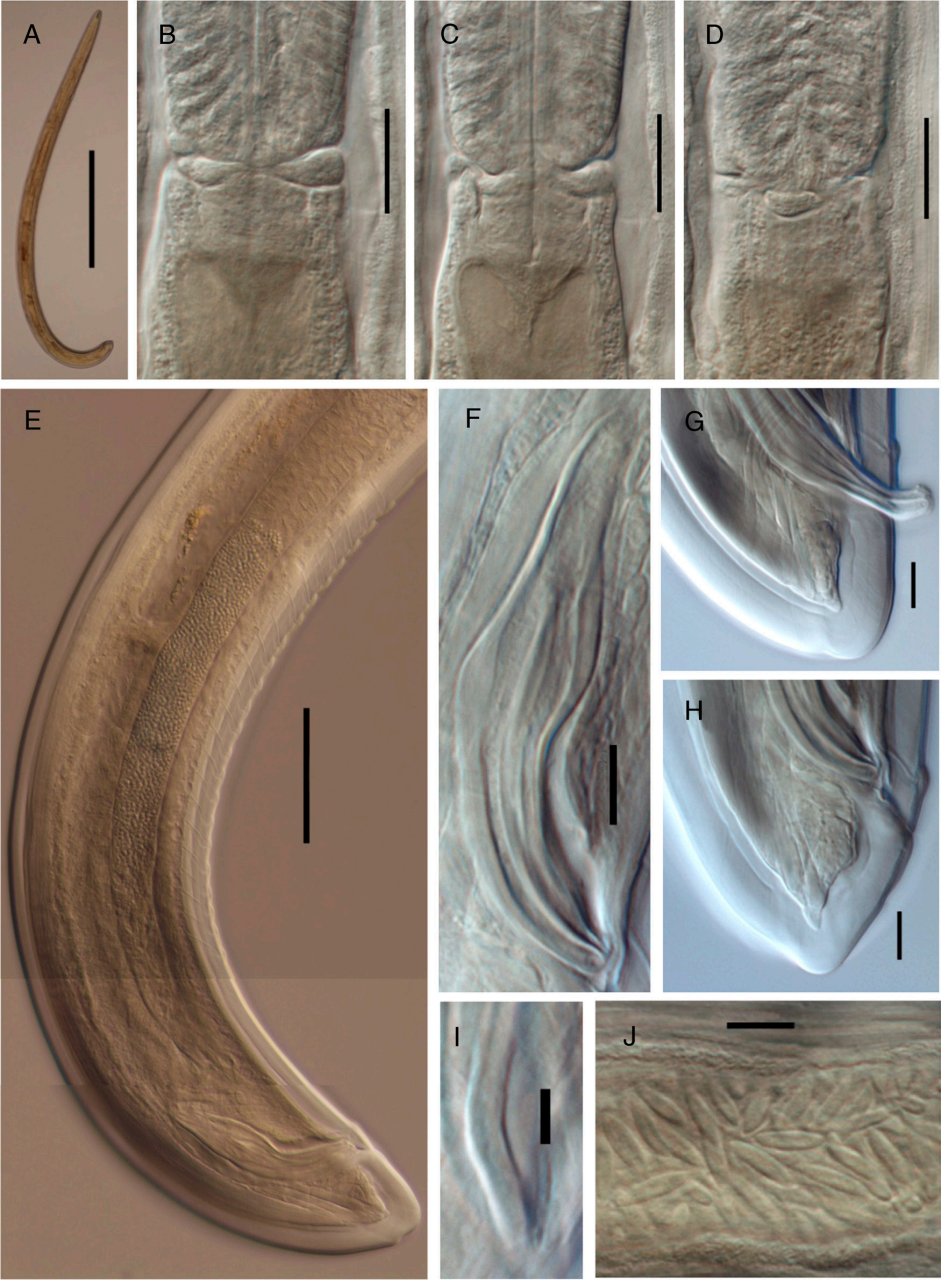
*Labronema montanum* sp. n. from Spain (male, LM). (A): Entire. (B–D): Pharyngo-intestinal junction showing the three lobes. (E): Posterior body region. (F): Spicule. (G, H): Male. (I): Lateral guiding piece. (J): Sperm cells. (Scale bars: A = 500 µm; D–B = 20 µm; E = 50 µm; F–H = 10 µm; I = 5 µm; J = 10 µm).

**Figure 4:**
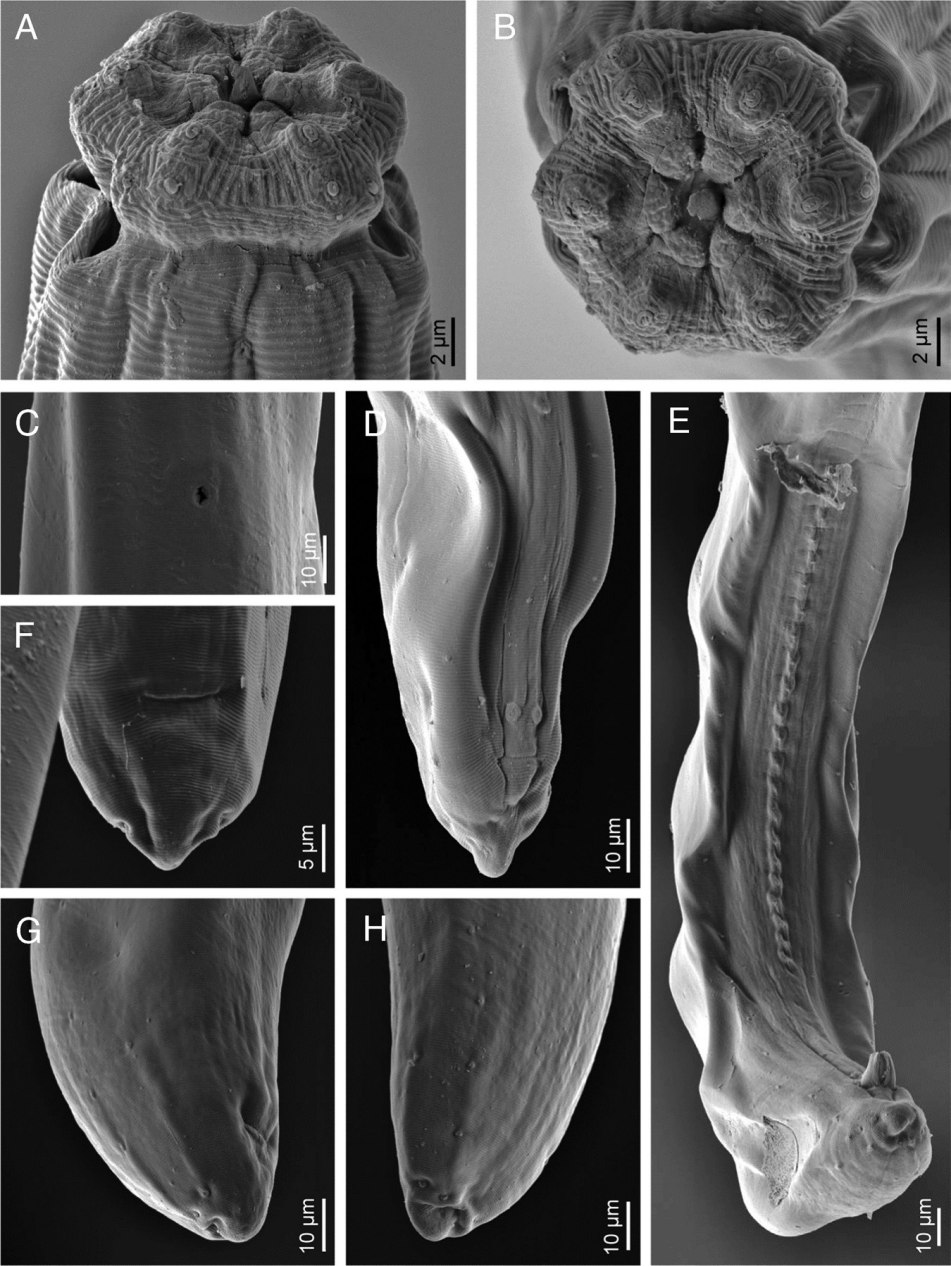
*Labronema montanum* sp. n. from Spain (SEM). (A): Lip region in ventral view. (B): Same in frontal view. (C): Vulva, ventral view. (D, E): Male, posterior body region showing ad-cloacal pair (D) and ventromedian series (E) of genital papillae. (F): Female tail, ventral view. (G, H): Female tail, lateral view, showing indentations of body cuticle.

### Material examined

In total, 17 females and 11 males, from one location, were examined.

### Morphometrics

See Table 1.

**Table 1.** Morphometrics of *Labronema montanum* sp. n. from Spain. Measurements in μm except L in mm, and in the form: average ± sd (range).

	Holotype	Paratypes	Paratypes
Character	♀	17♀♀	11♂♂
L	1.74	1.83 ± 0.14 (1.56–2.07)	1.87 ± 0.12 (1.68–2.08)
a	17.8	21.3 ± 2.0 (17.8–24.4)	22.7 ± 1.8 (20.3–25.9)
b	3.7	3.8 ± 0.3 (3.3–4.3)	3.7 ± 0.2 (3.3–4.1)
c	62	68.1 ± 9.4 (56–86)	72.2 ± 5.5 (65–84)
V	58	58.4 ± 1.2 (56.5–60.2)	–
c’	0.6	0.6 ± 0.1 (0.5–0.8)	0.6 ± 0.0 (0.6–0.7)
Lip region diameter	23	22.0 ± 1.1 (19–23)	22.2 ± 1.3 (19–24)
Odontostyle length ventral side	27	25.0 ± 1.9 (21–28)	26.0 ± 2.0 (22–29)
Odontostyle length dorsal side	28	26.5 ± 2.1 (23–30)	27.3 ± 2.0 (23–30)
Odontophore length	43	42.0 ± 1.9 (37–44)	42.7 ± 2.2(38–45)
Neck length	474	476 ± 27 (417–514)	500 ± 31 (450–551)
Pharyngeal expansion length	238	242 ± 19 (205–272)	247 ± 16 (212–264)
Body diam. at neck base	92	80.6 ± 8.3 (65–98)	78.9 ± 6.1 (72–91)
mid-body	98	85.9 ± 7.6 (72–99)	82.8 ± 8.9 (71–101)
anus/cloaca	44	43.9 ± 3.3 (39–51)	42.3 ± 3.7 (36–47)
Distance vulva – anterior end	1015	1068 ± 88 (889–1232)	–
Prerectum length	93	125 ± 23 (84–160)	202 ± 34 (150–268)
Rectum/cloaca length	61	60.0 ± 4.1 (52–68)	71.6 ± 5.6 (61–80)
Tail length	28	27.4 ± 4.5 (19–35)	26.1 ± 2.9 (20–30)
Spicules length	–	–	71.3 ± 3.6 (65–76)
Ventromedian supplements	–	–	(20–25)

### Description

#### Adult

Nematodes of medium size are stout to moderately slender (*a* = 18–26), with length 1.56–2.08 mm. The body is cylindrical, tapering toward both ends, but more so toward the anterior end, as the tail is short and rounded. Upon fixation, habitus visibly curved ventrad, more regularly in females, to an open C shape, and more perceptibly at posterior body region in males, to a J shape. Cuticle is three layered, especially obvious at caudal region, 3.5 to 6.5 µm thick at anterior region, 5.0 to 8.5 µm in mid-body, and 10.5 to 13.5 µm on female tail and 9 to 12 µm on male tail; outer layer is thin, with a very fine transverse striation (only appreciable in SEM images, Fig. 4A,B) and there is a constant thickness throughout the body; intermediate layer is much thicker than the outer one, particularly at the caudal region; inner layer is much thinner than the intermediate layer, especially distinct at the caudal region. The lateral chord is 10 to 22 µm broad, occupying 12 to 26% of mid-body diameter. Several ventral and dorsal pores are present at the cervical region. The lip region is offset by a deep constriction, 2.8 to 3.7 times broader than high and less than one-third (23–31%) of body diameter at the neck base. SEM observations are as follows: lips are mostly amalgamated, the lateral ones are barely differentiated from their adjacent subdorsal and subventral lips; papillae are button like with a pore at their center; labial papillae are surrounded by a circular striation that does not exist around the cephalic papillae; oral field is relatively wide, as the inner labial papillae are situated far from the oral aperture, bearing coarse radial incisures reaching the outer margin of the lip region, perioral area is differentiated in distinct but weakly protruding liplets. Amphid with funnel-shaped fovea is present; its aperture is 8 to 11 µm, occupying two-fifths to one-half (41–50%) of the lip region diameter. Cheilostom is nearly cylindrical, with thick walls, but it is lacking any other differentiation. Odontostyle is typical of the genus, strong, 5.3 to 7.1 times as long as broad, somewhat longer (1.1–1.3 times) than the lip region diameter, and 1.15 to 1.86% of total body length; aperture is 7 to 11 µm long, occupying one-third to two-fifths (33–42%) of the odontostyle length. Odontophore is rod-like; its length is 1.4 to 1.8 times the odontostyle. Pharynx is conspicuously muscular, with its slender portion enlarging very gradually and basal expansion being 5.1 to 7.9 times as long as wide, 2.5 to 3.6 times longer than body diameter at neck base, and occupying *ca* one-half (47–53%) of the neck length; pharyngeal gland nuclei are located as follows: DO = 46 to 55, DN = 49 to 58, S_1_N_1_ = 61 to 68, S_1_N_2_ = 69 to 78, S_2_N = 82 to 87, DN nucleolus 5 µm in diameter, S_1_N_1_ 2 µm and very close to its outlet, S_1_N_2_ 4 µm and at 3 µm from its outlet, and S_2_N 4.5 µm and at 9 µm from its outlet. The nerve ring is situated at 142 to 185 µm or 30 to 38% of the total neck length from the anterior end. Pharyngo-intestinal junction is well developed; it consists of conoid to conical, 25–52 × 16–26 µm, cardia, enveloped by the intestinal wall and a distinct ring-like structure protruding in three lobes that in some specimens show gland-like bodies (see remarks). Intestine is present with no relevant differentiation. The tail is short, convex conoid to rounded, often showing some kind of indentations, mainly on its dorsal side, probably as result of fixation process affecting the very thick cuticle at this region.

### Female

Genital system is amphidelphic, with both branches well and equally developed; the anterior is 207 to 399 µm or occupying 13 to 22% of the total body length; the posterior is 226 to 380 µm or 13 to 23% of the total body length. Ovaries are reflexed, variably sized; the anterior is 57 to 183 µm long, whereas the posterior is 72 to 158 µm long, usually reaching the oviduct–uterus junction, with oocytes first in two or more rows and then in a single row. Oviduct is 78 to 180 µm long or 1.0–2.3 times longer than the body diameter, consisting of a distal, slender section made of prismatic cells and a moderately developed proximal *pars dilatata* with visible lumen inside. A narrowing surrounded by a muscular ring (sphincter) separates oviduct and uterus. Uterus is 148 to 264 µm long or 1.7 to 3.5 times longer than the body diameter; it is tripartite, as it consists of distal, nearly spheroidal section with a visible lumen inside, a much slender intermediate, often convoluted, section with narrow lumen, and a proximal part with thin walls and a very wide lumen, nearly always containing abundant sperm cells inside. Vagina is extending inwards, 29 to 41 µm, to 36 to 46% of the body diameter: *pars proximalis* is 19–33 × 11–20 µm in size and it is surrounded by moderately developed circular musculature; *pars refringens* consists of (lateral view) either two small triangular, 4.5 to 5 µm long, sclerotized pieces, separated by a slightly less sclerotized intermediate area, or two large trapezoidal pieces, 5 to 7 µm long, in both cases with a combined width of 16 to 22 µm; *pars distalis* is 3.5 to 7.5 µm long. Vulva is a very short, *ca* 3 µm long, longitudinal slit or elliptical opening (Fig. 4C). Prerectum is 1.9 to 4.0 and rectum is 1.2 to 1.7 times longer than the anal body diameter.

#### Male

Prerectum is 3.2 to 7.4 and cloaca is 1.5 to 1.9 times longer than the body diameter at cloacal aperture. Genital system is diorchic, with opposed testes, and spindle-shaped sperm cells. In addition to the ad-cloacal pair, located at 8 to 11 µm from the cloacal aperture, there is a series of 20 to 25 contiguous, 5.5 to 11 µm apart, ventromedian supplements, ending in front of the anterior end of spicules, at 59 to 86 µm from the adcloacal pair; therefore, a distinct hiatus exists. Spicules are 4.6 to 5.8 times as long as wide, 1.4 to 1.9 times longer than body diameter at the cloacal aperture: head is 1.8 to 3.2 times longer than wide, occupying 31 to 36% of the total spicule length, and with slender walls, the dorsal one is longer than ventral and it is slightly curved; median piece is narrow, occupying hardly one-third (19–36%) of the maximum width of spicule, and reaching the posterior end, which is 4.5 to 6 µm broad; curvature is 130 to 140°; ventral hump and hollow are poorly demarcated but appreciable; the former is situated at 36 to 58% of the spicule length from its anterior end. Lateral guiding piece is curved ventrad, 19 to 25 µm long, 5.4 to 6.3 times longer than broad, and with somewhat bifid terminus.

#### Molecular characterization

**Figure 5:**
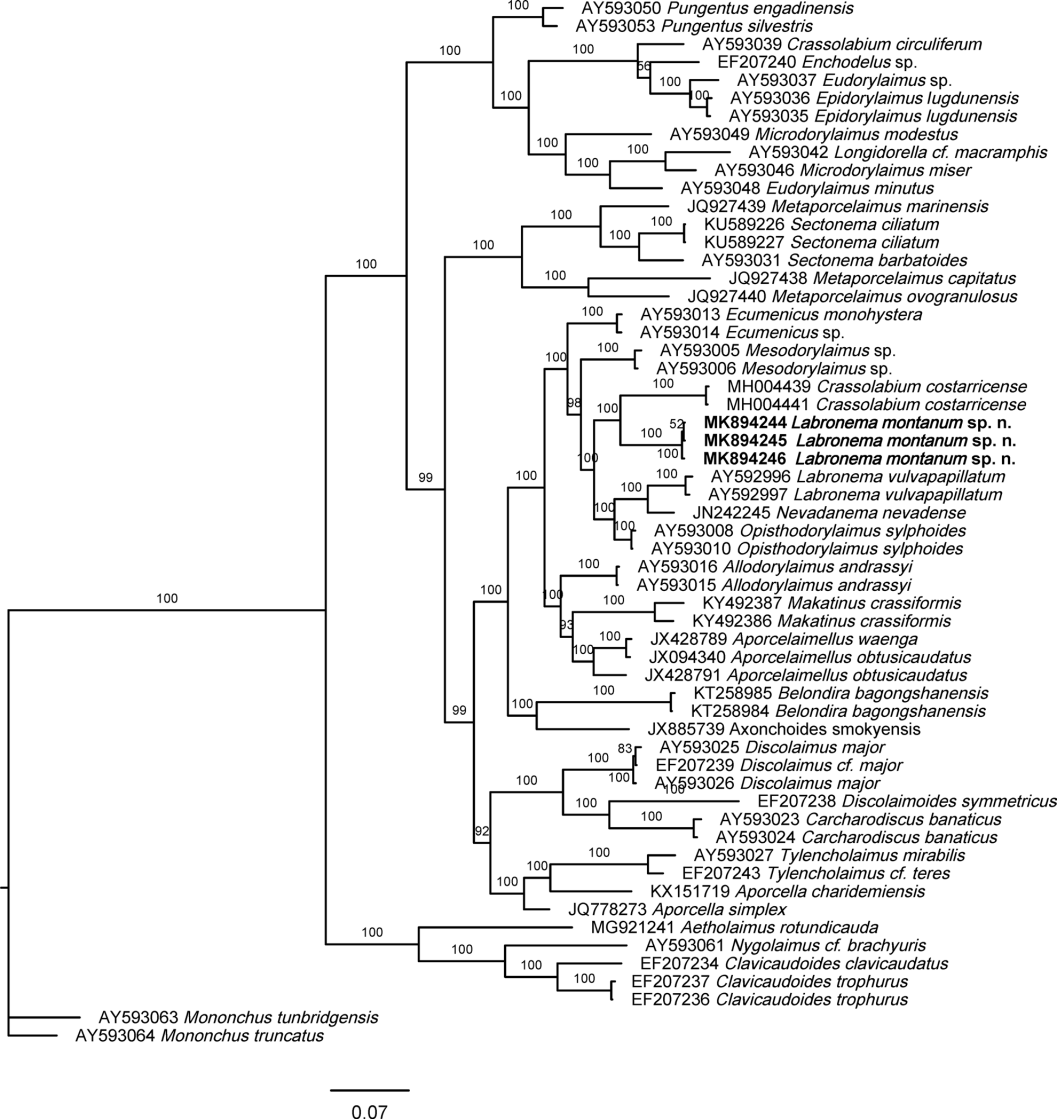
The Bayesian tree inferred from known and the newly sequenced *Labronema montanum* sp. n. based on sequences of the 28S rRNA region.

Three D2-D3 28S-rRNA sequences were obtained, one with 769, 772 and 783 pb long. These three fragments agree in 729 pb long, all of them were 100% identical. Their analysis has facilitated a study of the evolutionary relationships of the new species. The results are presented in Figure 5.

#### Diagnosis

The new species is characterized by its 1.56 to 2.08 mm long body, with lip region being offset by constriction and 19 to 24 µm broad, and with low perioral liplets, odontostyle 21 to 29 µm long at its ventral side, with aperture occupying 35 to 42% of its length, neck 417 to 551 µm long, pharyngeal expansion 205 to 272 µm long or occupying 47 to 53% of the total neck length, the presence of three cardiac lobes at the pharyngo-intestinal junction, didelphic–amphidelphic female genital system, a long and tripartite uterus, a longitudinal vulva (*V* = 57–60), short and rounded caudal region (19-35 µm, *c* = 56–86, *c’* = 0.5–0.8), spicules 65–76 µm long, and 20–25 nearly contiguous ventromedian supplements with hiatus.

#### Relationships

In its general morphology and morphometry, *L. montanum* sp. n. much resembles three Mediterranean species recently re-described by Peña-Santiago and Vinciguerra (2019), namely, *L. angeloi* Vinciguerra and Clausi, 1994, *L. carusoi* Vinciguerra & Orselli, 1998, and *L. duhouxi* (Altherr, 1963) Álvarez-Ortega & Peña-Santiago, 2013 (=*L. pulchrum* Vinciguerra and Zullini, 1980), from which it can be distinguished by small but significant differences. Thus, it differs from *L. angeloi* in its much thicker body cuticle (for instance, 10.5–13.5 vs 4–8.5 µm on female tail), broader lip region (19–23 vs 15–20 µm), longer neck (417–551 vs 386–402 µm), and pharyngeal expansion (205–272 vs 150–191 µm), the presence (vs absence) of three large lobes at pharyngo-intestinal junction, and a higher number of ventromedian supplements (20–25 vs 15–17). It differs from *L. carusoi* in its much thicker body cuticle (for instance, 10.5–13.5 vs 4.5–5.5 µm on female tail), longer neck (417–551 vs 342–382 µm), and pharyngeal expansion (205–272 vs 162–175 µm), the presence (vs absence) of three large lobes at pharyngo-intestinal junction, and longer spicules (65–76 vs 52–57 µm) with different morphology (large vs very small head). Also, it differs from *L. duhouxi* in its larger general size (body 1.56–2.08 vs 1.1–1.42 mm long, neck 417–551 vs 324–371 µm long, pharyngeal expansion 205–272 vs 142 µm), much longer spicules (65–76 vs 45–47 µm), and a higher number of ventromedian supplements (20–25 vs 15–16).

Evolutionary relationships, as derived from the analysis of D2-D3 28S-rRNA gene sequences, are presented in molecular tree of Figure 5. The new species forms part of a highly supported clade, predominantly constituted by representatives of the family Dorylaimidae, namely *Crassolabium, Labronema, Mesodorylaimus*, and *Nevadanema* sequences, and also by sequences of the genera *Ecumenicus* (Qudsianematidae) and *Opisthodorylaimus* (Thornenematidae). These results totally match recent previous findings (Varela-Benavides and Peña-Santiago, 2018), and confirm the present difficulties to elucidate the phylogeny of Dorylaimina. The internal relationships of the clade are not satisfactorily resolved, as the sequences corresponding to members of the subfamily Labronematinae (*Crassolabium, Labronema,* and *Nevadanema*) appear separated in two subclades, with both of them even including sequences of different species of the same genus, *Labronema*.

#### Type habitat and locality

The new species was collected from a grassy area with stony soil at 1,800 elevation in Pandera mountain, Jaén province, southern Iberian Peninsula, Spain.

#### Type material

Female holotype, 14 female paratypes and 7 male paratypes were deposited in the nematode collection of the University of Jaén, Spain. Two female paratypes and two male paratypes were deposited in the USDANC, Beltsville, Maryland, USA.

#### Etymology

The specific epithet is a Latin term that means belonging to the mountain, and it refers to type habitat where the new species was collected from.

#### Remarks

As mentioned above, the new species is similar to the three originally described in Italy, and together, they probably form a quite homogeneous group within the genus *Labronema* (cf. Peña-Santiago and Vinciguerra, 2019). They have been mainly reported in Mediterranean countries (Albania, Italy, Spain), and also in other neighboring territories (Romania and Switzerland).

The existence of three large lobes at the pharyngo-intestinal junction is a remarkable feature of the new species, already mentioned (as cardiac glands), in *L. pulchrum* (cf. Peña-Santiago and Vinciguerra, 2019). The present observations suggest that these lobes/glands are not comparable to those recognizable in nygolaims (suborder Nygolaimina). Actually, a more or less developed ring-like structure is easily perceptible in many Dorylaimina species, including *Labronema* representatives (for instance, *L. ferox* Thorne, 1930; see Peña-Santiago, 2019), which, in particular taxa, shows additional differentiation resulting in a large dorsal lobe, as observed in members of the genus *Aporcelinus* Andrássy, 2009, or three large lobes that apparently display a tri-radial symmetry.
